# Consumer perceptions and purchasing of packaged water products in Sierra Leone

**DOI:** 10.11604/pamj.2018.30.262.13676

**Published:** 2018-08-07

**Authors:** Mohamed Falilu Jalloh, Ashley Rhoderick Williams, Mohammad Bailor Jalloh, Paul Sengeh, George Saquee, Jamie Bartram

**Affiliations:** 1 FOCUS 1000, Freetown, Sierra Leone; 2 University of North Carolina at Chapel Hill, Chapel Hill, NC, United States

**Keywords:** Packaged water, Sierra Leone, consumer perceptions and behavior

## Abstract

**Introduction:**

Access to improved sources of drinking water remains a complex challenge in Sierra Leone and other low and middle income countries. We aimed to qualitatively examine consumer perceptions and purchasing behaviors of packaged water products in Sierra Leone.

**Methods:**

We conducted 25 focus groups with 178 consumers and petty traders of packaged water across the four geographic regions of Sierra Leone. Discussions were recorded, transcribed, and coded into themes. The Health Belief Model guided the thematic data analysis.

**Results:**

Packaged water was broadly perceived as safe, accessible, and convenient. Participants who lived outside of the capital city, Freetown, were more likely to report cost as a barrier. Personal experiences with a brand moderated trust levels. Self-reported handling behaviors of PW products were generally unhygienic. There was widespread belief that packaged water keeps newborn babies healthy. Consumers desired a simple mechanism to better identify government approved PW products.

**Conclusion:**

Perceived risks, benefits, barriers, self-efficacy, and reinforcing cues to action qualitatively influenced consumers’ purchasing behavior of packaged water. Government regulators should provide consumers with reliable means to identify approved packaged water products. Consumer education efforts should include hygienic handling of packaged water products in order to minimize post-production contamination.

## Introduction

In Sierra Leone, like most of sub-Saharan Africa, access to improved sources of drinking water remains a complex challenge; with water-related illnesses being a leading cause of death [1]. Almost 40% of Sierra Leoneans cannot access improved sources of drinking water [2]. Despite government efforts, Sierra Leone did not meet its Millennium Development Goal target to reduce the proportion of its population without improved access to drinking water by half between 1990 and 2015 [3, 4]. There has been a surge of packaged water (PW) businesses in the country, with an initial boom in the capital city of Freetown, and subsequent expansion in the provincial regions. The packaged water industry includes bottled water, machine-manufactured sachet water and handtied sachet water. Sachet water products are typically 500mL of water packaged either by machine using heat seal technology or by hand tying a plastic bag. Bottles and machine-manufactured sachets are often bundled together for wholesale but may also be sold individually in the market and in stores, while handtied sachets are usually sold individually. In Sierra Leone, most PW businesses produce machine manufactured sachet products, although a few large bottled water manufacturers exist in Freetown.

Despite the ubiquity of PW, the government lacks effective regulation to ensure its safety. PW is marketed as safe and free of contamination using slogans such as “pure drinking water” [5]. Contrary to these safety claims, 18% of PW products sampled at 49 manufacturing facilities in Freetown contained detectable Escherichia coli [6]. However, a systematic review revealed that while there are quality problems with PW, in some contexts, it is safer than the available alternative sources [7]. Post-production quality of PW tend to deteriorate along the supply chain. In Sierra Leone, one study found that the exterior of sachet products sold by street vendors were significantly more likely to contain E.coli and total coliforms as compared to those sold by retailers [6]. Even in the presence of enacted regulation, effective surveillance of the industry remains a challenge for government authorities [8]. Likewise, discarded packaging from PW poses an environmental challenge [9]. Consumers’ perceptions play a key role in their assessment of PW products and purchasing behaviors [10-13]. A study in Ghana revealed that sachet brands perceived to be of high quality were less likely to present detectable heterotrophic bacteria, after controlling for possible confounders, as compared to those perceived to be of low quality [12]. Another study in Nigeria found that the availability of a National Agency for Food and Drug Administration registration number influenced consumers’ purchasing decision, but that other labeling information rarely had any effect [11]. While prior studies have provided valuable insights on consumer perceptions of PW in sub-Saharan Africa, none of them grounded the findings using a theoretical framework.

Health behavior theories and models have been extensively used to understand consumption behaviors [14-17]. The Health Belief Model (HBM) asserts that an individual’s cognitive assessment of perceived risks, moderated by perceived benefits and barriers associated with a particular behavior, are reliable predictors of the behavior [14, 17]. Applying the HBM to PW consumption behavior suggests that consumers’ decisions could be influenced by their perceived susceptibility to waterborne contamination, perceived benefits and barriers of consuming PW, and cues to action such as labeling information, approval notices and branded messages. Using HBM as a guiding theoretical framework, the objective of this study was to qualitatively examine consumers’ perceptions that influence their purchasing behaviors of PW products. To the best of our knowledge, this is the first study that has employed a health behavior theory in examining the relationship between consumer perceptions and purchasing behaviors of PW products in sub-Saharan Africa.

## Methods

A total of 25 focus group discussions (FGDs) were conducted in Freetown (n = 10), Makeni (n = 5), Bo (n = 5) and Kenema (n = 5). These locations were purposively selected since they host a majority of PW businesses in the country and are geographically dispersed – including a mix of urban and rural areas. A nationwide survey conducted in 2013 found that over 90% of PW businesses were based in these locations [5]. In total, 178 PW consumers from the following homogeneous categories were selected for inclusion in the FGDs in July 2013: women aged 25 and above, men aged 25 and above, women aged 25 and above who were both consumers and petty traders of PW, men aged 25 and above who were both sellers and petty traders of PW, and mixed groups of male and female youth aged 18 - 24. We anticipated that heterogeneous groups would have been less likely to yield honest and vibrant conversations because men and elders within the groups may have dominated the discussions. Petty traders of PW were included in the FDGs as they represent an important segment of the PW supply chain, whose handling and storage practices of PW may have quality implications. Open-ended questions were developed around thematic areas guided by five HBM constructs: perceived susceptibility to waterborne contamination, perceived benefits of PW consumption, perceived barriers to PW consumption; perceived cues to action influencing PW purchasing behaviors, and self-efficacy to identify approved PW products. HBM’s perceived severity construct was not included in the thematic analysis since prior studies found it not to be a strong predictor of behavior adoption [14]. Twelve data collectors were trained to administer the FGDs through a two-day training workshop; and were divided into six teams, each comprising a facilitator and a note-taker. All data collectors were proficient in English and Krio – the commonly spoken local language in Sierra Leone. The FGD team members were also fluent in the indigenous language of their assigned locations. The FGD guide was pre-tested via three FGDs in Freetown. Minor revisions were then made in order to improve the clarity and sequencing of questions and probes.

**Figure 1:**
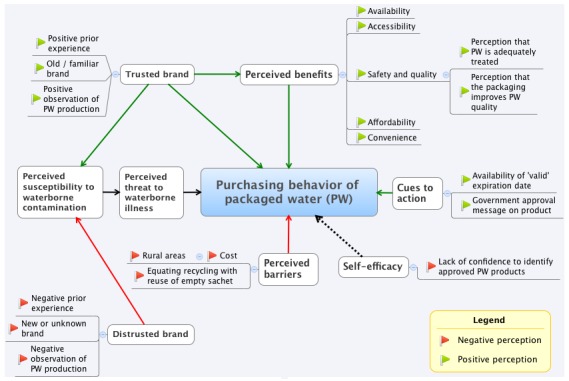
Mind map of consumer perceptions and purchasing behaviors of packaged water in Sierra Leone

Upon arriving at a locality, the teams approached potential participants to determine their eligibility to participate in the designated FGD category. The teams conducted FGDs in geographically dispersed areas within each locality. Each FGD started with an introduction of the data collection team and an explanation of the study. Written or thumb-printed informed consent was then obtained from the participants. The facilitator guided the conversations while the second team member took notes and tape-recorded the conversation. Non-verbal cues were noted. Discussions were mainly in Krio however some participants sporadically switched to Themne (in Makeni) or Mende (in Bo and Kenema) during the discussions. Recorded conversations were transcribed using ExpressScribe software. These transcripts were then imported into ATLAS.ti (ATLAS.ti Berlin, Germany). Two separate analysts reviewed and coded each transcript. Analysts then reviewed each other’s codes, discussed disagreements, and then harmonized the codes. An iterative review process ensued to identify emerging themes. Deviant themes and minority views were also identified. A mind map of the emerged themes was constructed using XMind software (Xmind Ltd. Hong Kong, China). The mind mapping was guided by HBM’s constructs – perceived susceptibility, perceived benefits, perceived barriers, cues to action and self-efficacy (Figure 1). The Institutional Review Board at the University of North Carolina – Chapel Hill (IRB #13-2165) and the Sierra Leone Ethics and Research Committee (WF2012V1) approved the study.

## Results

Most participants generally perceived themselves to be susceptible to waterborne illness, citing annual cholera outbreaks and frequent diarrhea in newborns and under-five children. Trusted PW brands were viewed as less likely to cause waterborne contamination as compared to the alternatives. Brands that had been on the market longer were more likely to be trusted as compared to new or unfamiliar brands. Consumers’ personal experiences with a brand also influenced their level of trust in that brand.

“Our well water used to be good but now it is no longer pure because children put harmful substances in the well. Rainwater is often polluted so I don’t drink it. I prefer to drink packaged water because it is inside a plastic, which is protected from cholera.” (Consumer, Makeni).

### Perceived benefits

PW was perceived to be easily accessible. Participants from Freetown reported having access to a wider variety of PW brands as compared to those outside of the capital. There was a widespread perception that most PW products are safe. Moreover, participant perceived that all PW have gone through proper treatment.

“I believe everybody likes something good. If according to what everybody is saying, that the package water is free from contamination, then I am happy to go for it not minding the cost. I usually can buy it at an affordable cost whenever I want it for myself and for my children.” (Consumer, Freetown).

Another recurring theme was the notion that trusted PW is not only safe for adults but could also help prevent diarrhea in newborns. Some participants mentioned that health care staff would usually advise them to use PW when preparing food for babies.

“Packaged water helps to stabilize a baby suffering from diarrhea and contributes to a speedy recovery. If you check in the hospital you will observe that packaged water is commonly used by all patients in the hospital. I believe packaged water is the best.” (Consumer, Makeni).

### Perceived barriers

While consumers in Freetown considered PW to be affordable, those in Bo, Kenema, and Makeni viewed cost as biggest barrier to their purchasing behaviors of PW. Consumers in Bo, Kenema and Makeni reported that they were more inclined to purchase local hand-tied sachets over other forms of PW. Hand-tied sachet water was seen as considerably more affordable. In Bo, Kenema, and Makeni communal wells were the main source of household water, including water for consumption. However, in the absence of cost barriers, consumers almost always preferred PW for consumption over alternatives.

### Cues to action

Participants who could read were more likely to pay attention to expiration dates as compared to other labeling information. While most consumers did not pay close attention to certification/licensing information, they found them reassuring when provided on the packages. When probed further, most participants explained that they have no means of verifying the statement provided on the sachets and bottles. Participants would like to have the ability to easily verify producers that have been approved by the relevant authorities.

### Self-efficacy

Most participants were not confident in their ability to properly objectively identify PW products that conformed to national quality standards. Across all FGDs, consumers expressed a desire for the government authorities to put mechanisms in place for the public to easily identify approved PW products. A common suggestion was for the government to publish a list of certified PW products in national newspapers and announce them on the radio. Participants would generally bite one of the edges and drink directly from the sachet or handtied sachet water. Few participants shared that they use a pair of scissors to cut the sachet and pour the water in a glass before drinking it. A majority of participants reported that they never wash their hands before handing PW products. However, they may use their hands or cloth to “wipe off” the sachet before biting into it. Consumers who were also petty traders of PW products reported similar handling behaviors.

## Discussion

The findings from the FGDs affirm the assertion of HBM that individuals are likely to engage in health related behaviors based on their weighing of perceived risks, benefits, barriers and self-efficacy to execute the behavior in the presence of reinforcing environmental cues [14]. Packaged water was viewed as more accessible than alternative sources of water for consumption – such as the municipal supply. Trust perceptions emerged as the factor that most frequently influenced consumers’ PW purchasing decision.

Consumer perceptions of PW products tended to be comparative in nature such that a consumer would evaluate the decision to drink PW against an alternative source. Even though the perceived safety of PW products reinforced purchasing decisions, the narrative is not straightforward. Safety perceptions were formed through intermediary factors such as prior experiences with a particular product and overall brand popularity – leading consumers to perceive products as either trustworthy or untrustworthy. The Theory of Planned Behavior (TPB), a value-expectancy theory, would suggest that PW use is a function of consumers’ intention to drink PW, with the attitudinal belief that it is safer than alternative sources, and normative belief that it is widely accepted by others in their social environment [15].

The HBM suggests that in order for an individual to engage in a desired behavior (e.g., purchase of only approved PW products), s/he would need to be confident in performing the promoted behavior [17]. The TBP supports this assertion as well by emphasizing that perceived behavioral control is often a reliable predictor of an individual’s engagement in a promoted behavior. Without a simplified means of verifying approved PW products, consumers would likely continue to make purchasing decisions based on their trust of PW products as opposed to objective safety measures – such as testing and approval by a sanctioned regulatory body.

The rapid urban expansion and deforestation in Sierra Leone [18, 19] render major constraints on municipal water sources such as the Guma Valley dam. Demand for improved sources of drinking water has increased, yet municipal sources are unable to meet this increased demand [20]. Therefore, the public often turns to PW for their drinking water. With a growing population depending on PW, there is a need for rigorous surveillance of PW production sites to ensure adherence to quality standards established by the Sierra Leone Standards Bureau in order to minimize the public health threat of waterborne disease outbreaks.

### Limitations

Despite efforts to standardize the administration and facilitation of the FGDs, a review of the transcripts revealed that there were minor variances in facilitation and probing techniques across some of the teams. However, we do not believe that these introduced systematic biases in responses of participants as questions were consistently posed in a neutral and open-ended manner. As with most qualitative studies, the sample size was small and only covered five out of 14 administrative districts in Sierra Leone. Furthermore, participants were purposefully selected for inclusion in the study. Nonetheless, the findings provide rich insights into better understanding the drivers and nuances of consumers’ perceptions and behaviors relating to an important industry that has exponentially grown in sub-Saharan Africa to meet the demand for safe drinking water.

## Conclusion

Our findings highlight the need for effective government regulation of the PW industry in Sierra Leone to ensure consumer protection. There is a need to provide consumers with simple means to identify approved PW products. Consumer and petty trader education should be an integral part of PW regulatory framework in Sierra Leone and other similar settings in the sub-Saharan region. A symbol linked to a government-generated list of approved PW products may be an effective way to communicate with consumers with no or low literacy. However, regulators would have to undertake rigorous surveillance to ensure that the approval symbol is not counterfeited or fraudulently used. Findings from the study further demonstrate the need to educate consumers and petty traders on hygienic handling of PW products in order to reduce the likelihood of post-production contamination.

### What is known about this topic

- Water-related illnesses are among the leading causes of death in Sierra Leone, especially among under-five children;
- Access to improved sources of drinking water remains a complex challenge in Sierra Leone and other parts of sub-Saharan Africa;
- There has been a surge of packaged water businesses in sub-Saharan Africa, however, regulation of the industry continues to be a challenge.

### What this study adds

- Widespread misconception that all packaged water products have been treated and are therefore safe for consumption;
- Most participants cannot distinguished approved packaged water products from those that have not been approved by government authorities;
- A recurring theme was for the government to institute simple means for consumers to verify approved PW products.

## Competing interests

The authors declare no competing interests.
